# Isolation and Characterization of a Primary Proximal Tubular Epithelial Cell Model from Human Kidney by CD10/CD13 Double Labeling

**DOI:** 10.1371/journal.pone.0066750

**Published:** 2013-06-14

**Authors:** Cynthia Van der Hauwaert, Grégoire Savary, Viviane Gnemmi, François Glowacki, Nicolas Pottier, Audrey Bouillez, Patrice Maboudou, Laurent Zini, Xavier Leroy, Christelle Cauffiez, Michaël Perrais, Sébastien Aubert

**Affiliations:** 1 EA4483, Département de Biochimie et Biologie Moléculaire, Faculté de Médecine de Lille, Pôle Recherche, Lille, France; 2 Institut National de la Santé et de la Recherche Médicale, U837, Centre de Recherche Jean-Pierre Aubert, Equipe 5 Mucines, Différentiation et Cancérogenèse Épithéliales, Lille, France; 3 Service d'Anatomie Pathologique, Centre de Biologie et Pathologie, CHRU Lille, Lille, France; 4 Faculté de Médecine de Lille, Université Lille 2, Lille, France; 5 Service de Néphrologie, Hôpital Huriez, CHRU Lille, Lille, France; 6 Service de Biochimie, Centre de Biologie et Pathologie, CHRU Lille, Lille, France; 7 Service d'Urologie, Hôpital Huriez, CHRU Lille, Lille, France; University of Tokushima, Japan

## Abstract

Renal proximal tubular epithelial cells play a central role in renal physiology and are among the cell types most sensitive to ischemia and xenobiotic nephrotoxicity. In order to investigate the molecular and cellular mechanisms underlying the pathophysiology of kidney injuries, a stable and well-characterized primary culture model of proximal tubular cells is required. An existing model of proximal tubular cells is hampered by the cellular heterogeneity of kidney; a method based on cell sorting for specific markers must therefore be developed. In this study, we present a primary culture model based on the mechanical and enzymatic dissociation of healthy tissue obtained from nephrectomy specimens. Renal epithelial cells were sorted using co-labeling for CD10 and CD13, two renal proximal tubular epithelial markers, by flow cytometry. Their purity, phenotypic stability and functional properties were evaluated over several passages. Our results demonstrate that CD10/CD13 double-positive cells constitute a pure, functional and stable proximal tubular epithelial cell population that displays proximal tubule markers and epithelial characteristics over the long term, whereas cells positive for either CD10 or CD13 alone appear to be heterogeneous. In conclusion, this study describes a method for establishing a robust renal proximal tubular epithelial cell model suitable for further experimentation.

## Introduction

The kidney, a key organ of the urinary system, plays a pivotal role in many physiological processes such as the maintenance of homeostasis, the excretion of nitrogen catabolism waste and the secretion of endocrine factors. In renal pathology and injury, all these processes are altered and accompanied by several symptoms: hypertension due to the alteration of the renin/angiotensin system and/or an imbalance of calcium and phosphorus metabolism induced by the deficiency of calcitriol [1].

Studying these pathophysiological mechanisms requires the use of *in vitro* models such as renal cell cultures. This methodology is limited by the complexity of the nephron, which consists of the glomerulus and various tubular segments (the proximal and distal tubules and collecting duct) and by the cellular heterogeneity of these segments, which comprise 15 types of epithelial cells with different properties and functions [2]. Among the different cell types, proximal tubular epithelial cells (PT cells) play a major role in the reabsorption of substances such as glucose and amino acids and the control of acid-base balance by the excretion of almost all the bicarbonate and the synthesis of ammonia [3]. They are also involved in the excretion of metabolic end products [4]. Furthermore PT cells are particularly sensitive to ischemic injury, and represent a primary target for xenobiotics, such as nephrotoxins (and their metabolites), whose effects can extend up to the kidney failure [5], [6].

To further elucidate the mechanisms of proximal tubular cell physiology and pathophysiology, as well as to study the potential mechanisms underlying nephrotoxins-induced renal toxicity, the primary culture of human proximal tubular cells represents a valuable tool [4], [7], [8].

Several techniques have been developed in order to establish such primary cultures: micro-dissection, enzymatic dissociation, the use of selective culture media, immunomagnetic cell sorting and isopycnic centrifugation [2], [4], [8]–[10]. However, only a few studies have verified the stability and differentiation status of these cells over time [2], [11]. In fact, one study has shown the likely transdifferentiation, and the loss of specific markers, of primary renal tubule cells such as human distal tubular epithelial cells [12].

The main goal of this work was therefore to develop primary cultures of human renal proximal tubular epithelial cells and to ensure the stability and differentiation status of these cells over several passages.

## Materials and Methods

### Ethics statement

This study was approved by the scientific committee of our institutional Biobank, Tumorothèque du CRRC de Lille (approval n°CSTMT100). For this non-interventional study, devoid of constitutional genetic characterization, only a verbal informed no-opposition for the use of tissue sample for research purpose is necessary according to the recommendations of the Haute Autorité de la Santé and the Code de la Santé Publique (Art L1211-2). This verbal consent was collected by the referring physician and notified on a special form in the patient record. For each surgical specimen, the absence of patient opposition was systematically verified and transmitted by the referring physician prior to the beginning of the cell isolation procedure. All tissue samples were de-indentified by the biobank.

### Cell isolation

The isolation of proximal tubular cells (PT cells) was performed as described by Helbert *et al*. (1997) [8] and Van der Biest *et al.* (1994) [13] with some modifications. Renal cortical tissue was collected from fresh nephrectomy specimens for renal or urinary tract cancer (n = 16). Mirror image samples of cortical tissue collected were analyzed by a pathologist to ensure the absence of cancer and significant parenchymal lesions. Cortical samples were decapsulated and dissected in order to obtain 1 mm^3^ fragments. The fragments were then digested in 6 mL of complete DMEM (Dulbecco's Modified Eagle's Medium)/F12 1∶1 medium (Invitrogen, Cergy Pontoise, France) containing 10 ng/mL Epidermal Growth Factor (EGF, Invitrogen), 1% penicillin/streptomycin (Invitrogen), 1% L-glutamine (Invitrogen), 15 mM HEPES (Sigma Aldrich, Saint Quentin Fallavier, France), 50 mM hydrocortisone (Sigma Aldrich), 5 µg/mL insulin (Invitrogen), 5 µg/mL transferrin (Sigma Aldrich) and 50 nM sodium selenite (Sigma Aldrich), with 2 mg/mL collagenase IV (enzymatic activity: 200 U/mL) (Invitrogen) and 20% dispase (Becton Dickinson, Le Pont de Claix, France). This step was performed three times at 37°C for 30 minutes, and the suspension was filtered through filters with a mesh size of 70 µm after each digestion. The cell suspension was washed twice in PBS (Phosphate Buffered Saline) and centrifuged for 5 minutes at 300 *g*. Cells were cultured in 75 cm^2^ plastic flasks with complete culture medium (DMEM/F12 1∶1 medium containing 10 ng/mL EGF, 1% penicillin/streptomycin, 1% L-glutamine, 15 mM HEPES, 50 mM hydrocortisone, 5 µg/mL insulin, 5 µg/mL transferrin and 50 nM sodium selenite) at 37°C under 5% CO_2_ in a humidified atmosphere. The culture medium was changed after 24 hours in order to eliminate non-adherent cells and residual cellular fragments.

### PT cell separation

PT cells were sorted using FACS (Fluorescence Activating Cell Sorting). Antibodies to two proximal tubular epithelial markers, CD10 and CD13 antibodies (eBioscience, Paris, France) were used [2], [8]. Confluent monolayers were washed twice with PBS and trypsinized for 5 minutes. One million cells were labeled with phycoerythrin (PE)-conjugated anti-CD13 or with allophycocyanin (APC)-conjugated anti-CD10 or both in 100 µL of complete medium (see cell isolation section). Antibody concentrations used were those described by the supplier. The cell suspension was incubated for 30 minutes at 4°C with the antibodies and washed in PBS. Cells were resuspended into 2 mL complete medium without EGF and with 0.5 mM EDTA, sorted using an Epic Altra cell sorter (Beckman Coulter, Villepinte, France) and collected in complete culture medium. Positively labeled cells were identified by their fluorescence when compared with that of appropriate control samples labeled using nonspecific isotype antibodies.

### Flow cytometry

CD10 and CD13 labeling of PT cells was performed on 0.5×10^6^ cells using the same conditions as for the FACS protocol, with a Cyan ADP analyzer (Beckman Coulter).

### Western blotting

Cells were lysed using a 25 mM Tris-HCl pH 7.5, 150 mM NaCl, 1% Sodium deoxycholate, 0.1% Sodium Dodecyl Sulfate buffer containing protease inhibitors (Roche, Meylan, France) and were sonicated for 20 seconds.

Total proteins were separated by electrophoresis on a 4–12% BisTris NuPAGE gel (Invitrogen) and were transferred onto nitrocellulose membrane using the iBlot system (Invitrogen). Western blotting was performed by incubating nitrocellulose membranes with specific primary antibodies overnight at 4°C. The following antibodies were used against: aquaporin-1 (clone B11; Santa Cruz, Heidelberg, Germany; 1∶100), N-cadherin (clone 3B9; Invitrogen; 1∶500), MUC1 (clone CT2; LabVison Corporation, Francheville, France; 1∶500), α-SMA (clone 4A8-2H3; Abnova, Le Perray en Yvelines, France; 1∶200) and β-actin (clone 13E5; Cell Signaling Technology, Saint Quentin Yvelines, France; 1∶1000). Membranes were incubated with secondary anti-rabbit, anti-Armenian hamster or anti-mouse antibodies coupled with horseradish peroxidase (Sigma Aldrich) for 45 minutes at room temperature. Immunoreactive bands were visualized by chemiluminescence using the Amersham ECL Plus Western Blotting Detection Reagent (GE Healthcare, Saclay, France) on a Molecular Imager ChemiDoc XRS System (Bio-Rad, Marnes-la-Coquette, France).

### Immunofluorescence

Cells were plated on Lab-Tek Chamber Slides (Fisher Scientific, Illkirch, France). Sub-confluent monolayers were fixed with 4% paraformaldehyde for 10 minutes. Fixed cells were washed with Tris Buffered Saline, Triton-X 100 0.1% (TBST) and incubated for 30 minutes in TBST-Bovine Serum Albumin 1% at room temperature, before incubating with primary antibodies overnight at 4°C. The following antibodies were used against: pan-cytokeratin (clone PCK-26; AbCam, Paris, France; 1∶200), β-catenin (clone 14 β-catenin; Beckton Dickinson; 1∶100), aquaporin-1 (1∶200), MUC1 (1∶200), E-cadherin (clone 36 E-cadherin; Beckton Dickinson; 1∶100), vimentin (clone LN6; Millipore, Molsheim, France; 1∶100) and α-SMA (clone 4A8-2H3; Abnova; 1∶100). Cells were incubated with secondary anti-mouse or anti-Armenian hamster antibodies coupled with Texas Red (Jackson Immunoresearch, Marseille, France) for 45 minutes at room temperature. Actin was labeled by incubation for 40 minutes with a phalloidin-FITC solution (Sigma Aldrich). Slides were mounted in Vectashield® HardSet^TM^ Mounting Medium with DAPI (Vector Laboratories, Les Ulis, France). Autofluorescence and non-specific fluorescence were measured on fixed control cells processed without antibodies and with the secondary antibody alone, respectively. Slides were examined under a fluorescence microscope (DM4000B, Leica, Germany).

### Transmission electronic microscopy (TEM)

Cells were grown in Boyden® chambers (Becton Dickinson) coated with Matrigel® (Becton Dickinson) or collagen IV (1 µg/cm^2^) (Becton Dickinson) or without matrix. Ten days after confluency, cells were fixed in paraformaldehyde 0.5% – glutaraldehyde 2%. A post-fixation step with OsO_4_ was performed. Membranes were dehydrated through an ethanol gradient and included overnight in Epon resin using the Epoxy Embedding Medium Kit (Sigma Aldrich). Ultra-thin sections were cut and stained with uranyl acetate and lead citrate to increase contrast. Sections were examined on an electronic microscope, Ziess EM902 (Carl Zeiss S.A.S, Le Pecq, France).

### Measurements of trans-epithelial electrical resistance (TEER)

Cells were seeded onto Boyden® chambers (Becton Dickinson) coated with Matrigel® (Becton Dickinson) or collagen IV (1 µg/cm^2^) (Becton Dickinson) or without matrix, at a density of 50 000 cells per well. At confluency, the TEER was measured for five days using a volt–ohmmeter Evom2^TM^ (World Precisions Instruments, Stevenage, United Kingdom) as following the manufacturer's instructions. Measurements were performed every 24 hours. The Madin-Darby Canine Kidney (MDCK) cell line (ECACC, Salisbury, United Kingdom) was used as a positive control [14]. TEER readings from empty uncoated or coated (Matrigel® or collagen IV) Boyden® chambers were subtracted from TEER readings obtained from similar chambers seeded with cells. TEER readings are expressed in Ω.cm^2^.

### Alkaline phosphatase assay

The expression of the proximal tubule brush border enzyme, alkaline phosphatase, was assessed spectrophotometrically using p-nitrophenylphosphatase as a substrate in agreement with the guidelines of the International Federation of Clinical Chemistry in the Cobas® 8000-2 robot (Roche). Enzyme activity was standardized to protein concentrations.

### Reverse transcriptase polymerase chain reaction (RT-PCR)

Total RNAs were extracted from CD10/CD13 double-positive and CD10/CD13 double-negative cells using MiRNeasy Mini Kit (Qiagen, Courtaboeuf, France) in accordance with the protocol described by the manufacturer, and concentrations were determined using the BioSpec-nano spectrophotometer (Shimadzu, Champs sur Marne, France). RT was performed using the High capacity cDNA reverse transcription kit (Applied Biosystems, Courtaboeuf, France) with manufacturer's instructions. Each amplification reaction was carried out in a total volume of 25 µL 10 mM Tris-HCl buffer pH 8.4 containing 50 mM KCl, 0.2 mM of each dNTP, 2 µM MgCl2, 0.4 µM of each primer (Table S1), 200 ng DNA and 0.6 U Taq DNA polymerase (Invitrogen). After an initial denaturation step at 94°C for 3 minutes, 35 cycles of 1 minute at 94°C, 1 minute of hybridization at 60°C and 1 minute of extension at 72°C were carried out. A final extension period of 7 minutes was performed at 72°C. RT-PCR products were loaded onto 1% agarose gels, stained with ethidium bromide and visualized under an UV-transilluminator.

### Statistical analysis

Data are presented as means ± SD. Continuous data were analyzed using a Student's *t*-test and a p- value <0.05 was considered to indicate a significant difference.

## Results

### Isolation of PT cells

Primary cultures were successfully established from sixteen fresh nephrectomy specimens.

**Figure 1:**
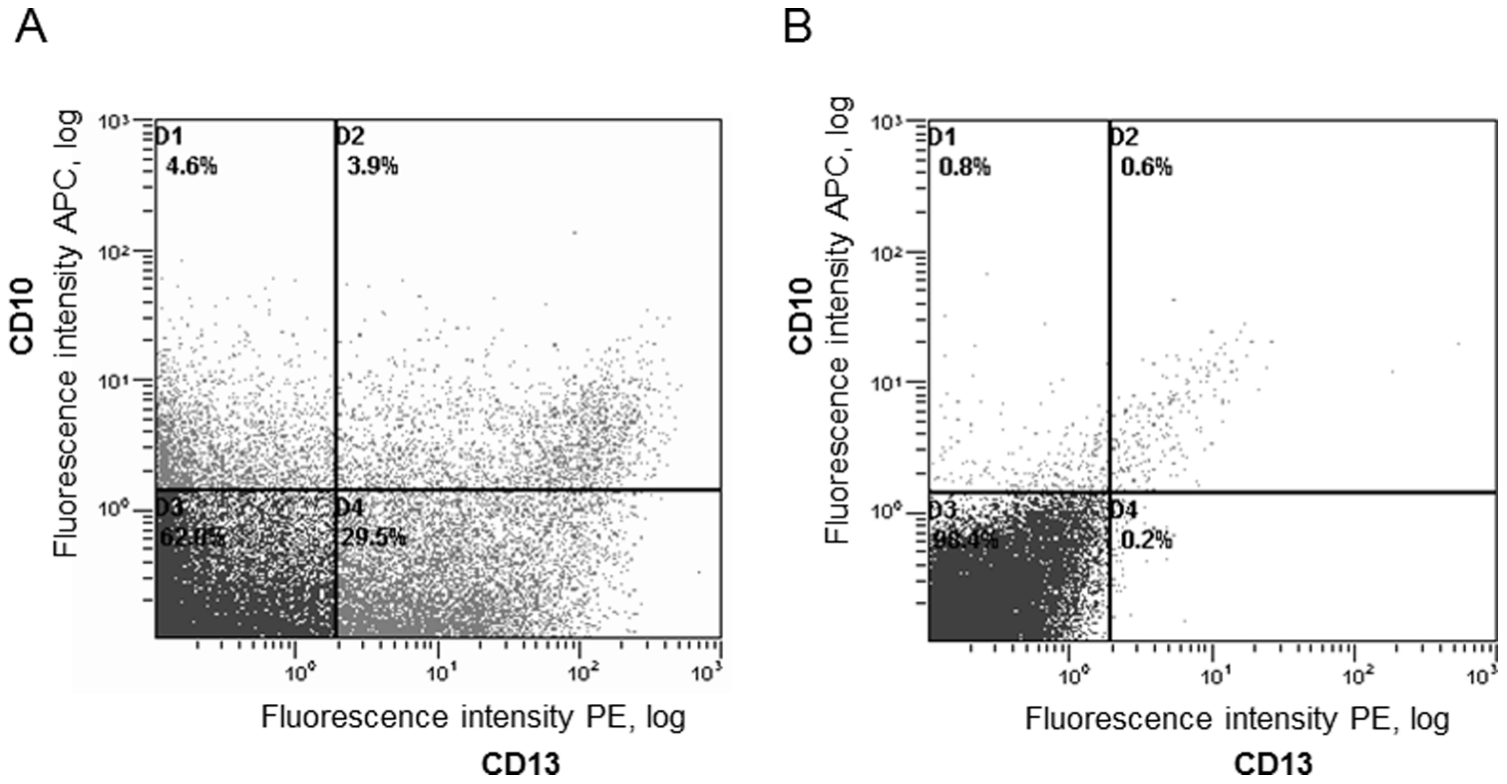
Sorting proximal tubular cells using specific antibodies. (A) Fluorescence plot showing cells labeled with antibodies against CD10 (APC: allophycocyanin) and CD13 (PE: phycoerythrin). FACS analysis revealed about 4% double-positive cells. (B) Fluorescence plot showing cells treated with isotypes to both antibodies to determine the upper threshold for non-specific fluorescence.

After 48 hours in culture, cultured renal cells exhibited the cobblestone-like appearance characteristic of epithelial cells (data not shown). Cells were isolated by FACS using antibodies to CD10 (neutral endopeptidase) and CD13 (aminopeptidase M), two markers previously described in PT cells [2], [8]. After cells sorting, the average percentage of CD10^+^, CD13^+^ and CD10/CD13 double positive cells, was about 8%, 33% and 4%, respectively (Figure 1A). The specificity of these antibodies was shown by isotype labeling (Figure 1B).

**Figure 2:**
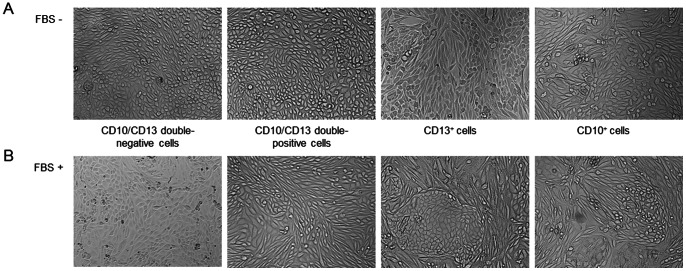
Representative morphology of primary CD10/CD13 double-negative cells, CD10/CD13 cells double-positive, CD13^+^ and CD10^+^cells. (A) Primary cultures at passage 2 in serum-free medium. (B) Primary cultures at passage 2 in medium with 10% FBS. Magnification: ×100.

When seeded onto plastic, cells formed monolayers and within two days, grew into small colonies. A few days after reaching confluency, the formation of domes characteristic of functional epithelial cells in culture was observed; these were more frequently noted in FBS-free EGF-supplemented medium [2] (Figure 2A).

The CD10^+^ and CD13^+^ cell populations appeared heterogeneous, with some cells exhibiting an epithelial morphology while others presented a more elongated appearance (Figure 2A).

In addition, different medium formulations were tested: FBS-free EGF-supplemented medium (Figure 2A) and FBS (10%)-supplemented medium without EGF (Figure 2B). In FBS-free medium, cells exhibited an epithelial morphology whereas in medium with FBS, cells exhibited an elongated fibroblast-like morphology (Figure 2B).

### Phenotypic characterization

**Figure 3:**
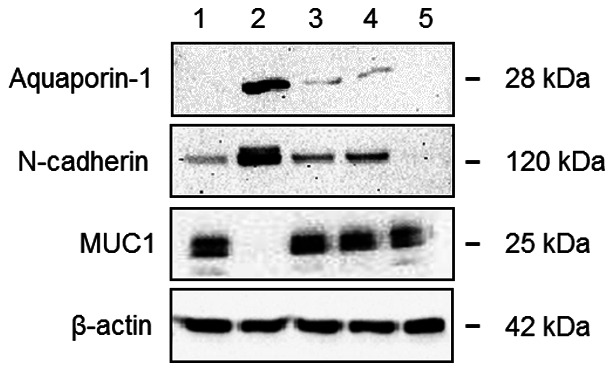
Expression of differentiation markers in different cell populations. Representative western blots for (1) unsorted cells, (2) CD10/CD13 double-positive cells, (3) CD10^+^ cells, (4) CD13^+^ cells and (5) CD10/CD13 double-negative cells. Blots were incubated with antibodies against aquaporin-1, N-cadherin, MUC1. The β-actin protein was used as an internal control. Proteins were extracted from cells at passage 2.

**Figure 4:**
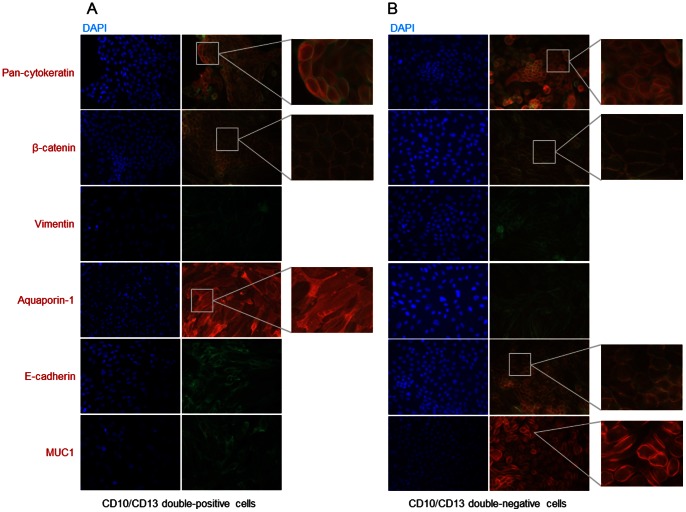
Immunofluorescence detection of specific markers in (A) CD10/CD13 double-positive cells and (B) CD10/CD13 double-negative cells. Actin was labeled by incubation with a phalloidin-FITC solution. Cells were labeled with antibodies to pan-cytokeratin, β-catenin, vimentin, aquaporin-1, E-cadherin and MUC1 (all Texas Red-conjugated). DAPI was used to counterstain nuclei. Magnification: ×200. The grey squares in the left panel images indicate the region of high magnification shown in the right panel. Experiments were performed with cells at different passages.

**Figure 5:**
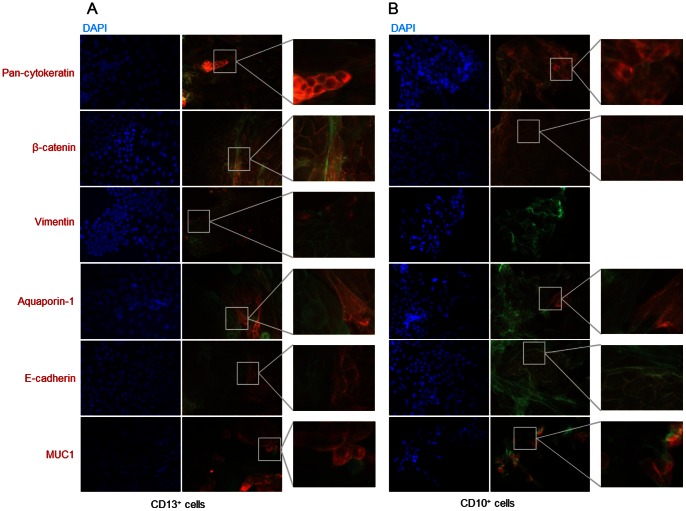
Immunofluorescence detection of specific markers in (A) CD13^+^ cells and (B) CD10^+^ cells. Actin was labeled by incubation with a phalloidin-FITC solution. Cells were labeled with antibodies to pan-cytokeratin, β-catenin, vimentin, aquaporin-1, E-cadherin and MUC1 (all Texas Red-conjugated). DAPI was used to counterstain nuclei. Magnification: ×200. The grey squares in the left panel images indicate the region of high magnification shown in the right panel. Experiments were performed with cells at different passages.

The initial characterization of sorted cells focused on detection of specific markers by western blotting. CD10/CD13 double-positive cells expressed the specific proximal tubule markers aquaporin-1 and N-cadherin, but did not express MUC1 (also known as epithelial membrane antigen), a specific distal tubule and collecting duct marker. By contrast, CD10/CD13 double-negative cells expressed MUC1 but not aquaporin-1 or N-cadherin (Figure 3). Similarly, immunofluorescence labeling revealed that CD10/CD13 double-positive cells were positive for aquaporin-1 but negative for MUC1 and E-cadherin, another distal tubule marker, contrarily to CD10/CD13 double-negative cells (Figure 4A and 4B respectively). Both cell populations expressed epithelial markers pan-cytokeratin and β-catenin at the cell membrane and were negative for the mesenchymal marker vimentin (Figure 4). By contrast, western blotting (Figure 3) and immunofluorescence labeling (Figure 5) of CD10^+^ or CD13^+^ cells alone revealed mixed populations that expressed both proximal and distal markers. Both cell populations also expressed epithelial markers (pan-cytokeratin and β-catenin at the cell membrane) (Figure 5). Also they were both positive for the mesenchymal marker vimentin when cultured in FBS-supplemented medium (data not shown) but negative for vimentin in FBS-free EGF-supplemented medium. Only a few cells of the CD13^+^-population displayed vimentin in both either culture medium (Figure 5A). Furthermore, unsorted cells, CD10^+^ cells, CD13^+^ cells, CD10/CD13 double-negative cells and CD10/CD13 double-positive cells expressed basal level of α-SMA by western blotting (Figure S1A). By contrast, no staining was detected by immunofluorescence (Figure S1B). These results are in agreement with many publications [15], [16] in which α-SMA staining was only detected by immunofluorescence after stimuli such as TGF-β, pharmacological or hypoxic treatments.

In summary, CD10/CD13 double-positive cells appeared to display the phenotypic characteristics of PT cells while CD10/CD13 double-negative cells presented the phenotypic characteristics of distal tubule and collecting duct cells.

### Morphological and functional characteristics

Since sorted cells presented a more epithelial phenotype in FBS-free EGF-supplemented medum than in FBS-supplemented medium, further characterization was carried out in serum-free medium. CD10/CD13 double-positive cells (PT cells) and CD10/CD13 double-negative cells were seeded onto Matrigel®, collagen IV or uncoated plastic membranes to evaluate the impact of the support on cell morphology and functional characteristics. Morphological and functional characteristics of single-labeled CD10^+^- and CD13^+^- cells were not evaluated since they expressed both proximal and distal markers.

**Figure 6:**
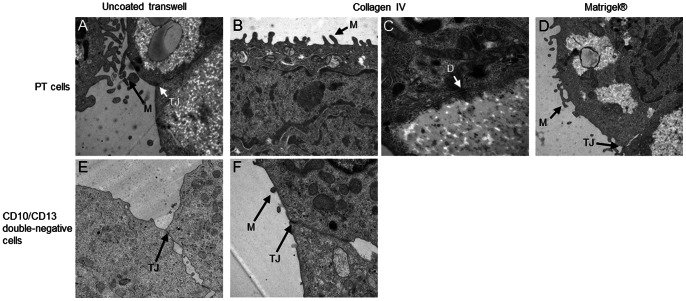
Ultrastructural morphology of cells. PT cells at passage 4 were seeded onto (A) uncoated transwell filters (×20 000), (B, C) collagen IV-coated filters (×20 000, ×140 000) and (D) Matrigel®-coated filters (×12 000). CD10/CD13 double-negative cells at passage 4 were seeded onto (E) uncoated transwell filters (×12 000) and (F) collagen IV-coated filters (×20 000). PT cells displayed a polarized morphology and exhibited tight junctions (TJ), long microvilli (M) and desmosomes (D). CD10/CD13 double-negative cells displayed a polarized morphology and exhibited tight junctions and short microvilli.

**Figure 7:**
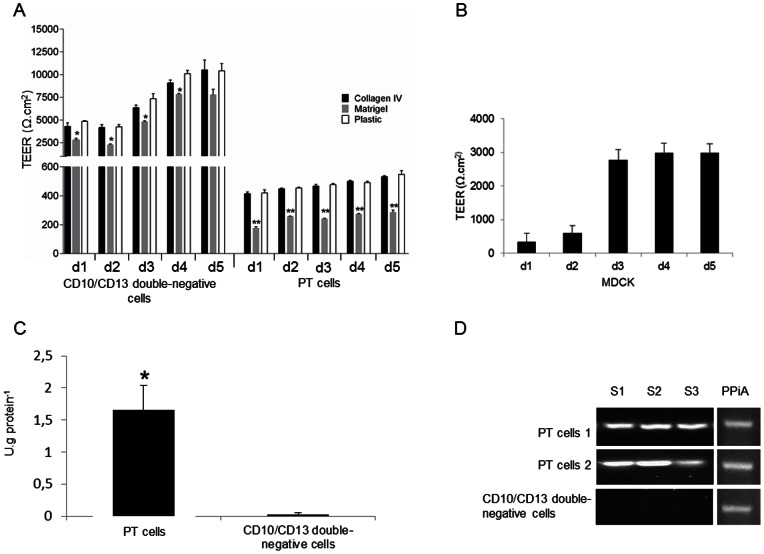
Functional characteristics of CD10/CD13 double-negative cells and PT cells. (A) Transepithelial electrical resistance (TEER) measurements were performed in CD10/CD13 double-negative cells and PT cells. Cells at passage 3 were seeded onto uncoated (plastic) transwell filters (white bar), collagen IV-coated filters (black bar) or Matrigel®-coated filters (grey bar). (B) The MDCK cell line was used as positive control, and cells were seeded onto uncoated transwell filters. The TEER was recorded at the points indicated (d: day). Means ± SD of three experiments are reported. *: p<0.05 and **: p<0.001 compared with similar results for cells seeded on plastic. (C) Alkaline phosphatase activity measured in PT cells at passage 5 and in CD10/CD13 double-negative cells at passage 5. Means ± SD of four experiments are reported. *: p<0.05 compared with CD10/CD13 double-negative. (D) Amplification of SGLT2 (S1 segment marker), CA IV (S2 segment marker) and SGLT1 (S3 segment marker) fragments in 2 representative PT cells and in CD10/CD13 double-negative cells at passage 3. Cyclophilin A (PPiA) was used as a house-keeping gene.

Ultrastructural analysis by TEM revealed that both PT cells and double-negative cells exhibited a polarized morphology with the occurrence of some tight junctions (more numerous in CD10/CD13 double-negative cells) as well as desmosomes, which are characteristics of polarized cells (Figure 6) [17]. Moreover, PT cells displayed long microvilli at the apical pole, indicative of a brush border [17]. By contrast, CD10/CD13 double-negative exhibited short microvilli. The type of support did not seem to influence cell morphology. In an attempt to evaluate epithelial barrier functionality, we measured the TEER across the PT cell- and CD10/CD13 double-negative cell-monolayers over 5 days post-confluency (Figure 7A). TEER values increased in a time-dependent manner as in MDCK control cells (Figure 7B) and were higher in CD10/CD13 double-negative cells than in PT cells. PT cells and CD10/CD13 double-negative cells grown on uncoated or collagen IV-coated transwell filters exhibited similar TEER values. However, TEER values were about two times lower for cells grown on Matrigel®-coated transwell filter (p<0.001 for PT cells and p<0.05 for CD10/CD13 double-negative cells) (Figure 7A). These results indicate that both plastic alone and collagen IV allow PT cells and CD10/CD13 double-negative cells to form a highly functional epithelium. Alkaline phosphatase activity, a proximal tubule brush border enzyme, was significantly higher in PT cells than in CD10/CD13 double-negative cells (p<0.05) (Figure 7C). In addition, RT-PCR showed that SLGT2, CA IV and SLGT1 mRNAs, specific of the S1, S2 and S3 segments respectively, were expressed in isolated PT cells (Figure 7D).

### Phenotypic stability over time

**Figure 8:**
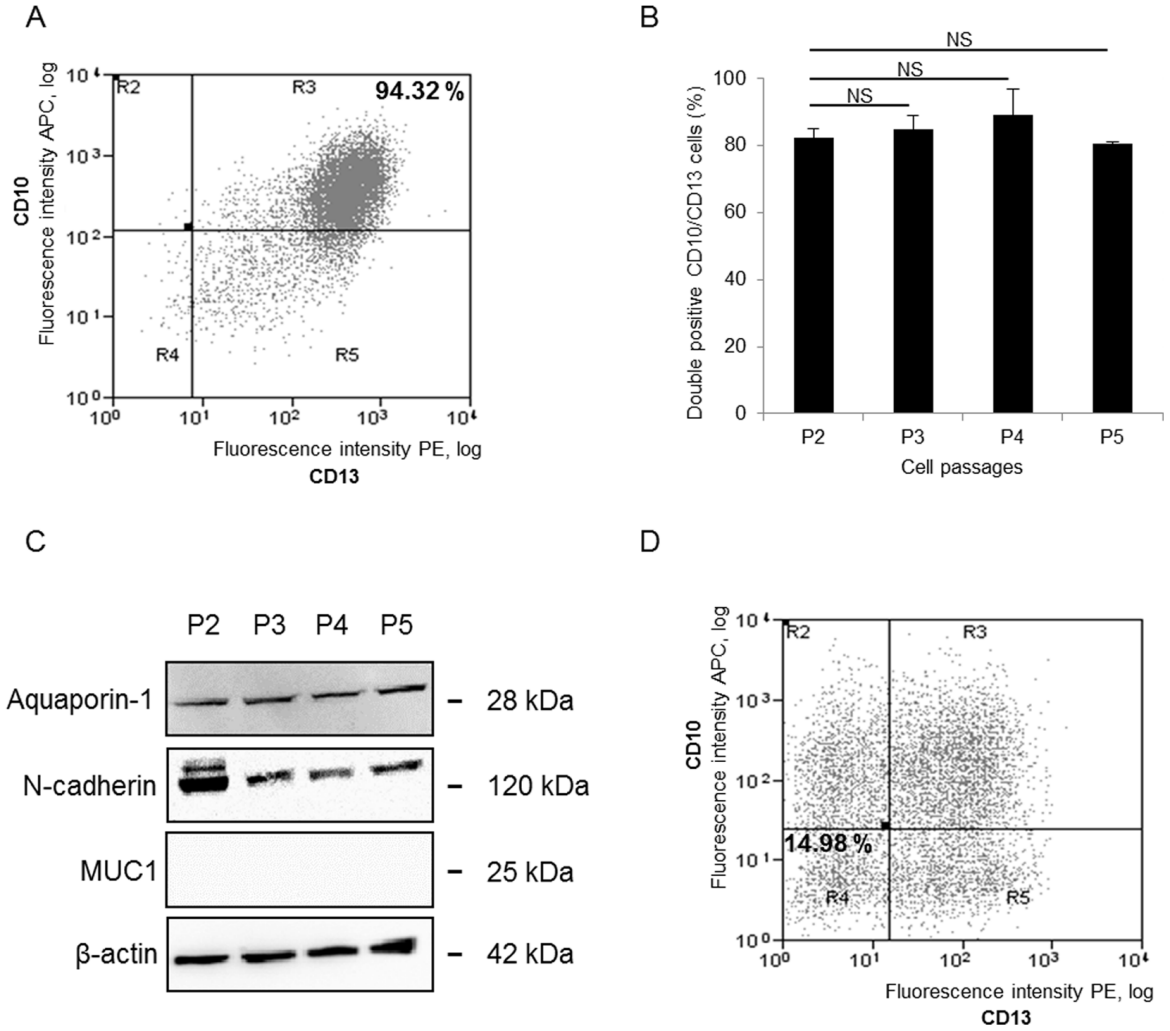
Evaluation of PT cells and CD10/CD13 double-negative cells phenotypic stability. (A) Fluorescence plot showing PT cells labeled with antibodies against CD10 (APC: allophycocyanin) and CD13 (PE: phycoerythrin) after four passages. Flow cytometry revealed about 94% double-positive cells. (B) Relative percentage of CD10/CD13 double-positive cells at passages 2, 3, 4 and 5 in the PT cells populations (n = 4). NS: non-significant (p>0.05). (C) Representative western blots for PT cells over 5 passages. Blots were incubated with antibodies against aquaporin-1, N-cadherin, MUC1. The β-actin protein was used as an internal control (D) Fluorescence plot showing the CD10/CD13 double-negative cell population labeled with antibodies against CD10 and CD13 after two passages. Flow cytometry revealed about 15% double-negative cells.

To determine the phenotypic stability of cell over time, we evaluated the expression of CD10 and CD13 over several passages. On flow cytometric analysis, PT cells positive for both CD10 and CD13 consistently exceeded 80% at passages 2, 3, 4 and 5 indicating that these cells are phenotypically stable at least over five passages (Figure 8 A–B). In addition, over five passages, aquaporin-1 and N-cadherin expression was persistent, and MUC1 was not expressed (Figure 8C). By contrast, only about 15% of CD10/CD13 cells that were initially double negative remained negative for both markers from the second cell passage onward (Figure 8D). This *de novo* expression of proximal tubule markers suggests the dedifferentiation of primary distal tubular epithelial cells in culture (Figure 8D).

## Discussion

Cell models such as renal cell lines and primary cultures are currently used for studies of renal physiology and nephrotoxicicity. Because of their immortalized status, renal epithelial cell lines such as HK-2 and HKC (two human proximal tubular cell lines) tend to dedifferentiate, lose their specific functions and to acquire non-tubule-specific characteristics [18], [19]. By contrast, primary cultured cells retain their phenotypic characteristics and specific functions such as hormonal responses, brush-border enzymatic activity and apical and basolateral transport systems, and are more representative of the *in vivo* human nephron at the physiological level [7], [8], [13], [20].

Since PT cells are common targets for xenobiotics due to their high transport activity, the primary culture of PT cells is an essential tool for studying nephrotoxicity [4], [21], [22]. The use of primary cell cultures allows these nephrotoxic mechanisms to be studied without the modification of metabolic processes that sometimes occurs in the existing immortalized cell lines. Indeed, previous studies have shown that primary cultures of rat, porcine and human PT cells exhibit a large spectrum of proteins involved in xenobiotic cellular processing. These cells express numerous biotransformation enzymes and drug transporters such as cytochromes P450, glutathione (GSH)-dependent enzymes, the ABC multidrug transporters and organic anion and cation transporters [4], [21]–[24]. In addition, primary cultures of human PT cells from multiple donors allow the *in vitro* study inter-individual variations in cellular metabolic capacity. Primary human PT cell cultures are commercially available and have been used in several toxicological studies [5], [25], [26]. However, in spite of the interest of these models, they present several disadvantages, such as a unique donor for each lot. Moreover, when tested by flow cytometry, commercial PT cells displayed lower expression levels of both CD10 and CD13 in our hands (Figure S2), suggesting a heterogeneous renal epithelial population. In fact, the study of responses that are consistent across individuals could be hampered by the use of these commercial models.

Although several studies have previously described protocols for establishing PT cell primary cultures, these are hampered by frequent heterocellular contamination, cellular differentiation/dedifferentiation and poor viability [10]. The aim of our work was to establish and characterize a model of primary PT cells that would ensure their phenotypic purity and the stability, as well as verify its limitations. In our study, we used a FACS protocol for the isolation of a highly differentiated population of PT cells. Since previous studies have shown that surface markers can be used for flow cytometric selection [20], we used two specific proximal tubular epithelial cell surface markers CD10 and CD13 [2], [8]. We demonstrate that populations that express either CD10 and CD13 alone are in fact mixed populations expressing both proximal and distal tubule markers, although several studies have based their models of primary human PT cell cultures on cell sorting for CD13 alone [2], [27]. By contrast, CD10/CD13 double-positive cells express only proximal markers (aquaporin-1 and N-cadherin) while double-negative cells express only distal and collecting duct markers (MUC1 and E-cadherin). Moreover, we confirm the previous demonstration by Sens *et al* (1999) [28] that renal cells exhibit an epithelial phenotype when cultured in FBS-free medium supplemented with EGF. Our results thus support the view that only CD10/CD13 double-positive sorted cells cultured in serum-free medium with EGF represent a pure population of proximal tubular epithelial cells.

About 4% of our sorted cells were double-positive for CD10 and CD13, and corresponded to PT cells. Even though this yield is rather low, the FACS method possesses the great advantage of being highly specific and allowing a highly purified cell population to be obtained [13], [20]. Furthermore, in comparison with immunomagnetic separation, FACS allows double-labeled cells to be sorted directly.

To ensure that the sorted PT and double-negative cells were fully epithelial and functional, further characterization was carried out. As shown by TEM, whatever the matrix used (plastic, collagen IV or Matrigel®), PT cells and CD10/CD13 double-negative cells displayed a characteristic epithelial morphology with long and short microvilli respectively, as well as tight junctions and desmosomes. Tight junctions play a critical role not only in epithelial barrier function, but also in ion, protein and small molecule transport. Furthermore, tight junctions and desmosomes participate in the baso-apical polarity of cells [29]. The TEER also provides an assessment of the presence of tight junctions, and thus of monolayer integrity; as well as polarity [29]. Indeed, CD10/CD13 double-negative cells exhibit more tight junctions and a higher TEER than PT cells, as previously reported [30]. Since, to our knowledge, no study has as yet investigated the impact of the matrix on the TEER of renal cells. Matrigel® was used to mimic the basal lamina. Surprisingly, PT cells on Matrigel® did not display sufficient resistance, as though they were unable to form a completely tight layer on this matrix. This is quite similar to the findings of Delabarre *et al* (1997) using mammary cells [31]. To further characterize PT cells functionality, phosphatase alkaline activity (a proximal tubule brush border enzyme [11], [12]) was measured and was significantly higher in PT cells than in CD10/CD13 double-negative cells. These results, consistent with previous reports [2], [4], [30], [32]–[34], support the view that monolayer of cells was functional. Structurally, the proximal tubule consists of three segments: S1 (the early convoluted tubule), S2 (the end of the convoluted tubule) and S3 (the straight proximal tubule) [35]–[37]. By evaluating expression of SLGT2, CA IV and SLGT1 at mRNA levels, specific markers of the S1, S2 and S3 segments respectively [27], [34], [38], our results indicated that CD10/CD13 double-positive cells express markers of all segments of the proximal tubule. To validate our model of PT cells, we ensured its phenotypic stability over time by flow cytometric assay and western blotting on five passages since at passage 6, PT cells lost their proliferation capacity. Indeed, the PT cell phenotype was preserved at least until the fifth cell passage, and their dedifferentiation rate was quite low when compared to CD10/CD13 double-negative cells, which displayed the *de novo* expression of CD10 and CD13. This phenomenon has been previously described [12], and highlights the difficulty of carrying out pathophysiological studies on primary renal distal tubular epithelial cells.

In conclusion, we have established a model of primary human PT cells using a FACS protocol based on CD10/CD13 double labeling. These highly purified primary cultured cells retained their specific characteristics in EGF-supplemented medium on plastic over several cell passages. Our model could be a useful tool for studies focusing on the pathophysiology of human renal proximal tubule.

## Supporting Information

Figure S1 **Expression of α-SMA in different cell populations.** (A) Representative immunoblotting of (1) unsorted cells, (2) CD10+ cells, (3) CD13+ cells, (4) CD10/CD13 double-negative cells, (5) PT cells at passage 2, (6) PT cells at passage 3, (7) PT cells at passage 4 and (8) PT cells at passage 5. Blots were incubated with antibody against α-SMA. The β-actin protein was used as an internal control. (B) Immunofluorescence detection of α-SMA (antibody Texas Red-conjugated) in PT cells and in MRC5 cells, a fibroblastic cell line exposed to TGF-β, used as a positive control. Cells were labeled by incubation with a phalloidin-FITC solution. DAPI was used to counterstain nuclei. Magnification: ×200.
(TIF)

Figure S2 **Phenotypic analysis of commercial PT cells.** Fluorescence plot showing commercial PT cells (from ScienCell Research Laboratories, Nanterre, France) labeled with antibodies against CD10 (APC: allophycocyanin) and CD13 (PE: phycoerythrin) after three passages. Flow cytometry revealed about 42% double-positive cells.
(TIF)

Table S1 **Summary of forward and reverse primers used to generate PCR products.**
(DOC)
