# Supplementary figures and images for: Isolation and Characterization of a Primary Proximal Tubular Epithelial Cell Model from Human Kidney by CD10/CD13 Double Labeling
Source: PLoS One. 2013 Jun 14;8(6):e66750. doi: 10.1371/journal.pone.0066750 (PMC3682988; doi:10.1371/journal.pone.0066750)

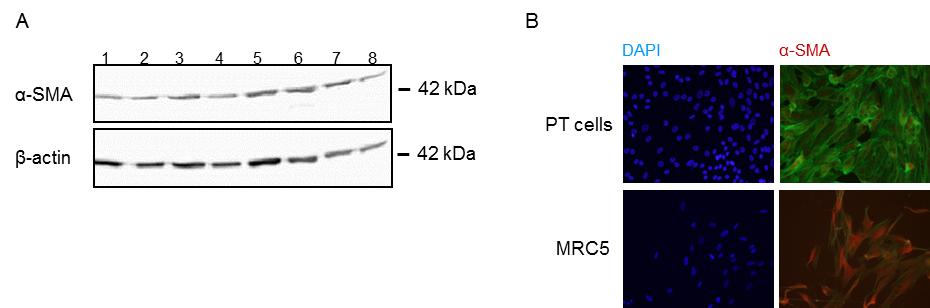

Supplement: Figure S1 — Expression of α-SMA in different cell populations. (A) Representative immunoblotting of (1) unsorted cells, (2) CD10+ cells, (3) CD13+ cells, (4) CD10/CD13 double-negative cells, (5) PT cells at passage 2, (6) PT cells at passage 3, (7) PT cells at passage 4 and (8) PT cells at passage 5. Blots were incubated with antibody against α-SMA. The β-actin protein was used as an internal control. (B) Immunofluorescence detection of α-SMA (antibody Texas Red-conjugated) in PT cells and in MRC5 cells, a fibroblastic cell line exposed to TGF-β, used as a positive control. Cells were labeled by incubation with a phalloidin-FITC solution. DAPI was used to counterstain nuclei. Magnification: ×200. (TIF) [file pone.0066750.s001.tif]

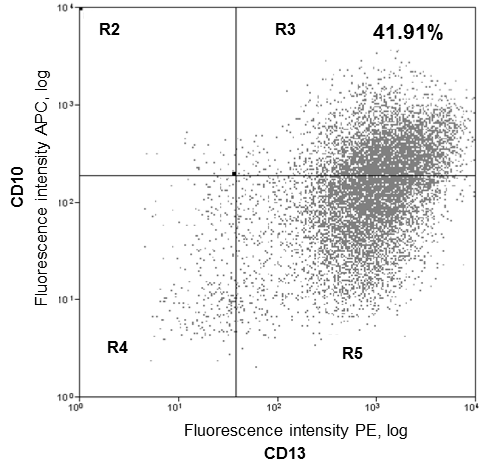

Supplement: Figure S2 — Phenotypic analysis of commercial PT cells. Fluorescence plot showing commercial PT cells (from ScienCell Research Laboratories, Nanterre, France) labeled with antibodies against CD10 (APC: allophycocyanin) and CD13 (PE: phycoerythrin) after three passages. Flow cytometry revealed about 42% double-positive cells. (TIF) [file pone.0066750.s002.tif]
