# Supplementary material for: Isolation and Characterization of a Primary Proximal Tubular Epithelial Cell Model from Human Kidney by CD10/CD13 Double Labeling
Source: PLoS One. 2013 Jun 14;8(6):e66750. doi: 10.1371/journal.pone.0066750 (PMC3682988; doi:10.1371/journal.pone.0066750)
Supplement: Table S1 — Summary of forward and reverse primers used to generate PCR products. (DOC) [file pone.0066750.s003.doc]

**Table S1. Summary of forward and reverse primers used to generate PCR products.**

| **Target** | **Primer (forward 5’-3’)** | **Primer (reverse 5’-3’)** |
| --- | --- | --- |
| PPiA | TCCTGGCATCTTGTCCAT | TGCTGGTCTTGCCATTCCT |
| SGLT1 | CCTCTTCGCCATTTCTTTCATC | ATGCACATCCGGAATGGGT |
| SGLT2 | TTCAGTCTCCGGCATAGCAA | CATCTCCATGGCACTCTCTGG |
| CA IV | CTTCATCCTCGTCGGCTATG | ATGGCAAAGTGTCTCC |
